# Jiang Tang Xiao Ke Granule, a Classic Chinese Herbal Formula, Improves the Effect of Metformin on Lipid and Glucose Metabolism in Diabetic Mice

**DOI:** 10.1155/2016/1592731

**Published:** 2016-06-21

**Authors:** Yi Zhang, Hong An, Si-Yuan Pan, Dan-Dan Zhao, Jia-Cheng Zuo, Xiao-Ke Li, Ya Gao, Qian-Qian Mu, Na Yu, Yue Ma, Fang-Fang Mo, Si-Hua Gao

**Affiliations:** ^1^Basic Theory of Chinese Medicine, Preclinical Medicine School, Beijing University of Chinese Medicine, Beijing 100029, China; ^2^Department of Pharmacology, School of Chinese Materia Medica, Beijing University of Chinese Medicine, Beijing 100102, China; ^3^Diabetes Research Center, Beijing University of Chinese Medicine, Beijing 100029, China

## Abstract

In the present study, the hypoglycemic, hypolipidemic, and antioxidative effects of metformin (MET) combined with Jiang Tang Xiao Ke (JTXK) granule derived from the “Di Huang Tang” were evaluated in mice with type 2 diabetes mellitus (DM) induced by high-fat diet/streptozotocin. DM mice were orally treated with MET (0.19 g/kg) either alone or combined with different doses (1.75, 3.5, or 7 g/kg) of JTXK for 4 weeks. Results showed that the serum and hepatic glucose, lipids, and oxidative stress levels were elevated in DM mice, when compared with the normal mice. MET treatment decreased FBG and serum glucagon levels of DM mice. Combination treatment with MET and JTXK 3.5 g/kg increased the hypoglycemia and insulin sensitivity at 4 weeks when compared with the DM mice treated with MET alone. However, neither MET nor MET/JTXK treatment could completely reverse the hyperglycemia in DM mice. JTXK enhanced the serum triglyceride (TG) and hepatic lipid-lowering effect of MET in a dose-dependent manner in DM mice. JTXK 1.75 and 3.5 g/kg improved the hepatoprotective effect of MET in DM mice. Synergistic effect of combination treatment with MET and JTXK on antioxidant stress was also found in DM mice compared with MET alone.

## 1. Introduction

Diabetes mellitus (DM) is a metabolic disease with decreased glucose transport into muscle and fat cells and increased hepatic glucose output resulting from dysfunction in insulin secretion or resistance to its activity [1, 2]. DM characterized by chronic hyperglycemia and multiple complications affects millions of individuals worldwide nowadays [3]. One of the most common complications of DM is nonalcoholic fatty liver disease (NAFLD), a risk factor for the development of type 2 diabetes [4, 5]. NAFLD is considered as the hepatic manifestation of the metabolic syndrome caused by abnormal accumulation of triglyceride (TG) inside the hepatocytes [6, 7]. NAFLD induced lipotoxicity is particularly germane to the liver and can lead to apoptosis referred to as lipoapoptosis [8, 9], nonalcoholic steatohepatitis (NASH), fibrosis, and cirrhosis [5, 10]. In the recent past, both experimental and clinical data have demonstrated that oxidative stress is involved in DM and DM secondary complications such as NAFLD and NASH [11–13]. Oxidative stress can lead to mitochondrial dysfunction, endoplasmic reticulum stress, and insulin resistance, all of which could lead to DM ultimately [14]. Oxidative stress and diminished antioxidants within the liver are the key features of NAFLD/NASH while insulin resistance is largely responsible for the development of NAFLD/NASH, which causes hepatic steatosis [15].

Currently, there are 4 kinds of available drugs for the treatment of DM, including *α*-glucosidase inhibitors, biguanides, sulphonylureas, and thiazolidinediones [16]. However, some of these antidiabetic agents are noted to have adverse side effects such as gastrointestinal disturbances and hypoglycemia [17]. Metformin (MET) is a currently available oral antidiabetic/hypoglycemic agent to deal with patients with type 2 diabetes. It can lower blood glucose and positively affect lipid profiles associated with few clinically deleterious adverse events [18]. More than two thousand years ago, herbal remedies had been widely used by traditional Chinese medicine (TCM), a major oriental healthcare system, practitioners for the prevention, and treatment of various diseases in China [19–21]. Up to now, the herbal remedies have shown universal adjustment in the treatment of DM by lowering blood glucose [22] and mitigating its related complications such as lipid metabolic disorders [23, 24], including NAFLD associated with lipid metabolism [25, 26]. TCM have been attracting more and more attentions for their complementary therapeutic effects to western medicine [27, 28] and overall adjustment. It has been demonstrated that several medicinal plants recorded in traditional Chinese pharmacopeia have antidiabetic effect. For example,* Coptis chinensis* and* Astragalus membranaceus* were found to improve insulin resistance for Type 2 DM [29–31].

For more than 30 years, professor Gao has been probed to use medicinal plants for treating DM. Based on the clinical experience, laboratory research, and classic herbal formula, he has created Jiang Tang Xiao Ke (JTXK) granule which lowered blood glucose and improved insulin resistance in patient and/or animals with DM [32, 33]. In this study, we aimed to assess the antidiabetic and hypolipidemic effect of MET combined with JTXK granule on glucose and lipid profiles, as well as the oxidative stress parameters in both the serum and liver tissues in DM mice.

## 2. Materials and Methods

### 2.1. Preparation Procedure of JTXK Granule

**Figure 1:**
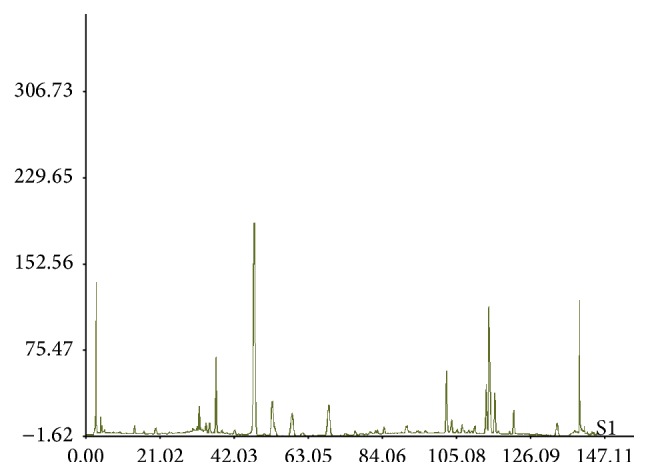
Fingerprinting of JTXK granule.

Chinese herbal medicine* Radix Rehmanniae* (Di Huang),* Radix Salviae Miltiorrhizae* (Dan Shen),* Fructus Corni* (Shan Yu Rou),* Panax Ginseng* (Ren Shen), and* Rhizoma Coptidis* (Huang Lian) at the proportion of 3 : 3 : 1 : 1 : 1 were used to prepare JTXK granule. The raw herbs were purchased from Beijing Tong Ren Tang medicinal materials Co., Ltd., Beijing, China, and authenticated by Professor Chun-Sheng Liu in the Beijing University of Chinese Medicine. 4.5 kg of dried tuber of* Radix Rehmanniae* and* Radix Salviae Miltiorrhizae* was boiled thrice with 12 volumes of distilled water for 1 hour. The pooled extract was filtered and concentrated by rotary evaporator at 40°C until the relative density reached 1.15. 1.5 kg of dried* Fructus Corni*,* Panax Ginseng*, and* Rhizoma Coptidis* was extracted thrice with 12 volumes of 60% (v/v, in H_2_O) ethanol under reflux after soaking for half an hour. The pooled ethanolic extract was filtered and concentrated by above-mentioned method. Every gram (wet weight) of ethanolic extract was equivalent to 1 g of dried herbs. JTXK granule was prepared using the mixture of both aqueous extract and ethanolic extract and stored at 4°C until use [32]. One gram of JTXK was equivalent to 5 g of crude herbs. Fingerprinting of JTXK granule was shown in Figure 1.

### 2.2. Chemicals and Regents

MET was purchased from Tianjin Yabao pharmacy Co., Ltd. (Tianjin, China). Streptozotocin (STZ, Cat. number SLBB7526V) was obtained from Sigma Aldrich Chemical Co., Ltd. (St. Louis, USA). STZ was dissolved into 0.1 mol/L and pH 4.5 sodium citrate hydrochloric acid buffer when needed. Blood glucose kit and triglyceride (TG) kit were obtained from Beijing Leadman Biochemical Co., Ltd. (Beijing, China). Assay kits for total cholesterol (TC), low-density lipoprotein cholesterol (LDL-C), high-density lipoprotein cholesterol (HDL-C), superoxide dismutase (SOD), malondialdehyde (MDA), and total glutathione (GSH) were obtained from Zhongsheng Beikong Biotechnology and Science Inc. (Beijing, China). Insulin ELISA assay kit and glucagon kit were obtained from Beijing North Biotechnology Research Institute (Beijing, China).

### 2.3. Animals and Treatment

Crl:CD1 (Institute of Cancer Research) outbred male mice, weighing 42 ± 3 g, were supplied by Vital River Lab Animal Co., Ltd. (Beijing, China). Animals were kept on a 12 h light/dark cycle and at 24 ± 2°C, with the humidity of 55 ± 5%. Mice were allowed to adapt to the environment for one week before experiment. All animal studies were performed according to protocols approved by the Institutional Animal Care and Use Committee of Beijing University of Chinese Medicine, China.

### 2.4. Experimental Design

#### 2.4.1. Diabetic Mouse Model

Six mice were randomly assigned to normal control group and given standard diet, and the other 40 mice were fed with high-fat diet (HFD) containing 20% sucrose (w/w), 2.5% cholesterol (w/w), 10% lard (w/w), and 0.3% sodium cholic acid (w/w) in standard feed, which was provided by Ke'ao Xieli Feed Co., Ltd. (Beijing, China). Mice were fed with HFD for 4 weeks and intraperitoneally injected with STZ 100 mg/kg to induce DM. The mice with fasting blood glucose (FBG) ≥ 11.1 mmol·L^−1^ were confirmed as having diabetes at 72 h after STZ treatment [34].

#### 2.4.2. DM Mice Treatment

According to the FBG level and weight of each mouse, DM mice were randomly divided into 5 groups of 6 animals in each: (1) drug-untreated DM mice; (2) DM mice treated with MET 0.19 g/kg; (3), (4), and (5) DM mice treated with MET 0.19 g/kg plus JTXK granules 1.75, 3.5, and 7 g/kg, respectively. Both MET and JTXK granule suspended in water were administered by gavage for 4 weeks. Weekly, body weights were recorded for all groups. After fasting for 12 h, mice were sacrificed under light ether anesthesia. Whole blood samples obtained from the abdominal aorta were centrifuged for 15 min at 3,000 rpm/min to obtain the serum. Serum samples were stored at −20°C until biochemical analyses. Liver were dissected out of mice, and hepatic weight and hepatic index (liver weight/body weight × 100) were measured and then washed in ice-cold 0.9% NaCl solution for biochemical and histopathological analysis.

### 2.5. Oral Glucose Tolerance Test (OGTT)

Mice were fasted overnight. The next morning, glucose 2 g/kg was gavaged into the fasten mice. Glucose levels of blood sample from tail vein of mice with a tail-incision technique were estimated by using glucometer at 0, 30, 60, and 120 min.

### 2.6. Blood/Serum Analysis

At 0, 2, and 4 weeks after the drug administration, the FBG from the tail vein were monitored by glucometer (Johnson & Johnson). The fasting serum insulin (FINS) and glucagon levels were determined according to the instruction of kits. Serum total cholesterol (TC), triglyceride (TG), high-density lipoprotein (HDL), low-density lipoprotein (LDL), alanine aminotransferase (ALT), aspartate aminotransferase (AST), malondialdehyde (MDA) contents, and superoxide dismutase (SOD) activity were measured with the relevant kits and methods according to the manufacturer's protocols.

### 2.7. Liver Biochemical Analysis

Liver were cut into small pieces and homogenized on ice with corresponding buffer (1 : 9, w/v). The homogenates were centrifuged at 3000 rpm for 15 min at 4°C and the supernatants were used to determine the TG, TC, HDL-C, LDL-C, and MDA contents and SOD and glutathione (GSH) activities according to the manufacturer's protocols of different commercial kits. Protein of the liver homogenate was estimated by BCA protein quantitative analysis kit.

### 2.8. Liver Histological Evaluation

Liver were fixed in 10% neutral buffered formalin, embedded in paraffin, sectioned at 4-5 *μ*m by rotary microtome, stained with hematoxylin-eosin, and examined by laboratory microscopy (Olympus, Tokyo, Japan) to assess the histopathological changes.

### 2.9. Statistical Analysis

All values are expressed as means ± SD of the mean. Data were analyzed by one-way analysis of variance (ANOVA) using SPSS (version 17.0) statistical analysis program, and then differences among means were analyzed using Dunnett's multiple comparisons test or post hoc analysis. Differences were considered significant at *p* < 0.05.

## 3. Results

### 3.1. Effect of MET Combined with JTXK on Serum Glucose Levels in DM Mice

In this study, the FBG levels in DM mice were approximately 5-fold higher than that in normal mice. Compared with the DM mice, the FBG values in MET (0.19 g/kg) treatment alone had a trend of reduction (by 18.15%) after 2 weeks of treatment but did not reach statistical difference (*p* > 0.05). Significant difference (*p* < 0.01) was observed at 4 weeks after MET treatment. However, the same dose of MET combined with JTXK 1.75, 3.5, and 7 g/kg lowered FBG levels by 25.21, 36.63, and 37.81%, respectively, at 2 weeks after MET/JTXK treatment, when compared with DM group mice, but had no statistical differences (*p* > 0.05) in comparison with mice treated with MET alone. The FBG levels were lowered by 41.04, 57.78, and 48.75%, respectively, in the mice treated with same dose of MET combined with JTXK 1.75, 3.5, and 7 g/kg at 4 weeks after treatment, when compared with the untreated DM mice. Compared with MET alone group, MET combined with JTXK 3.5 g/kg treatment can significantly reduce the level of FBG in DM mice (Table 1).

### 3.2. Effect of MET Combined with JTXK on Serum OGTT Levels in DM Mice

OGTT is an efficient way to assess the insulin secretion induced by glucose taken and glycemic control. After 4-week treatment with MET and MET/JTXK, DM mice were gavaged with glucose (2 g/kg body weight). Both normal and untreated DM mice received the same values of vehicle. Blood glucose levels were measured from the tail vein at 0, 30, 60, and 120 min after glucose treatment. Results showed that blood glucose was significantly decreased (up to 57.57%) in the drug-treated DM mice compared with the drug-untreated DM mice at 120 min after glucose taken. However, MET combined with JTXK 3.5 g/kg, but not 1.75 and 7 g/kg, increased the MET-induced hypoglycemia (by 38.37%) compared with MET alone (Table 2).

### 3.3. Effect of MET Combined with JTXK on Serum FINS and Glucagon Levels in DM Mice

**Figure 2:**
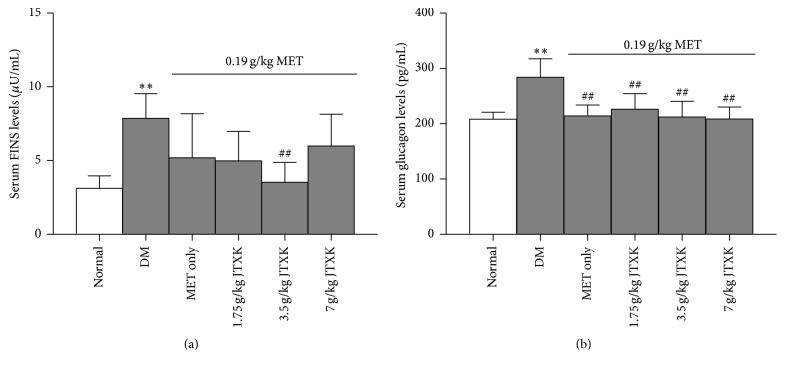
Effect of MET combined with JTXK on serum FINS and glucagon levels in DM mice. ^*∗∗*^ *p* < 0.01 versus the normal group; ^##^ *p* < 0.01 versus the DM group.

After 4-week treatment, the serum FINS and glucagon levels were determined. Results showed that both serum FINS and glucagon levels of DM mice were much higher (by 60.36 and 26.70%, resp.) than those of normal mice. Although MET treatment alone did not reduced the FINS levels, combination of MET and JTXK 3.5 g/kg treatment significantly lowered FINS levels (up to 55.15%), when compared with the drug-untreated DM mice. A lower level of serum glucagon was found in the DM mice treated with MET, which was not affected by the combination of JTXK (Figure 2).

### 3.4. Effect of MET Combined with JTXK on Serum Lipids in DM Mice

The serum TG, TC, and LDL levels in DM mice were markedly increased by 434, 235, and 316%, respectively, compared with the normal mice. However, serum HDL levels were decreased by 43.83% in DM mice. MET treatment for 4 weeks markedly reduced serum TG and TC levels (by 38.69 and 36.61%, resp.), but it increased serum HDL level (by 57.99%), when compared with the drug-untreated DM mice. JTXK treatment enhanced the TG-, TC-, and LDL-lowering effect of MET (up to 38.57, 27.22, and 15.01%, resp.) in a dose-dependent manner. The combination of MET and JTXK 1.75 g/kg elevated the serum HDL level by 25.90% compared with the MET alone (Table 3).

### 3.5. Effect of MET Combined with JTXK on Hepatic Lipids in DM Mice

**Figure 3:**
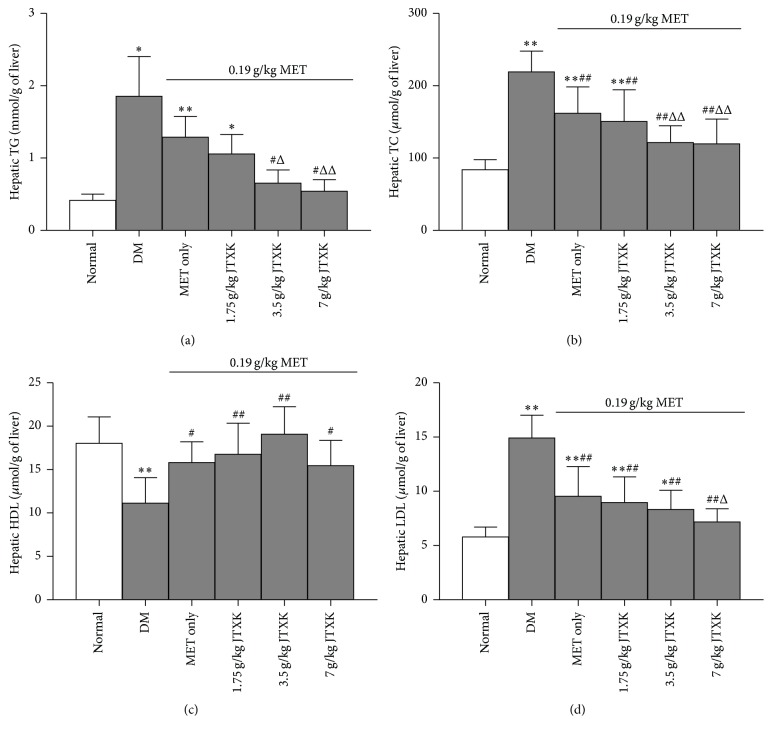
Effect of MET combined with JTXK on hepatic lipids in DM mice. ^*∗*^ *p* < 0.05 and ^*∗∗*^ *p* < 0.01 versus the normal group; ^#^ *p* < 0.05 and ^##^ *p* < 0.01 versus the drug-untreated DM group; ^Δ^ *p* < 0.05 and ^ΔΔ^ *p* < 0.01 versus the MET alone group.

Figure 3 showed that DM mice developed a significant increase in hepatic TG (up to 367%), TC (up to 154%), and LDL (up to 157%) levels and a marked decrease in HDL (by 38.23%) level in liver homogenate when compared with those of normal mice. Treatment with MET significantly reduced hepatic TC and LDL contents (by 23.85 and 35.98%, resp.), but it increased HDL contents (by 41.87%) in DM mice. JTXK enhanced the effect of hepatic TG-, TC-, and LDL-lowering effect by MET (up to 70.82, 21.91, and 24.85%, resp.) in a dose-dependent manner. Nevertheless JTXK did not affect the alteration of MET on hepatic HDL contents.

### 3.6. Effect of MET Combined with JTXK on Hepatic Function and Mass in DM Mice

As shown in Table 4, a significant increase in serum ALT and AST (by 227 and 225%, resp.) levels was observed in DM mice, when compared with those of normal mice. Treatment with MET for 4 weeks decreased the serum ALT (by 33.71%) and AST (by 37.68%) activities in DM mice. Treating DM mice with MET plus JTXK (1.75 and 3.5 g/kg) significantly reduced ALT (up to 31.99%) and AST (up to 24.60%) activities, when compared with MET alone. However, MET combined with JTXK 7 g/kg raised the serum ALT and AST levels (by 28.03 and 42.80%, resp.) in comparison with the DM mice treated with MET alone. Hepatomegaly was found in DM mice. MET treatment lowered the hepatic mass by 22.51% in DM mice compared with the untreated DM mice. JTXK treatment dose-dependently extended the hepatic mass-lowering effect of MET.

### 3.7. Effect of MET Combined with JTXK on Oxidative Stress in DM Mice

**Figure 4:**
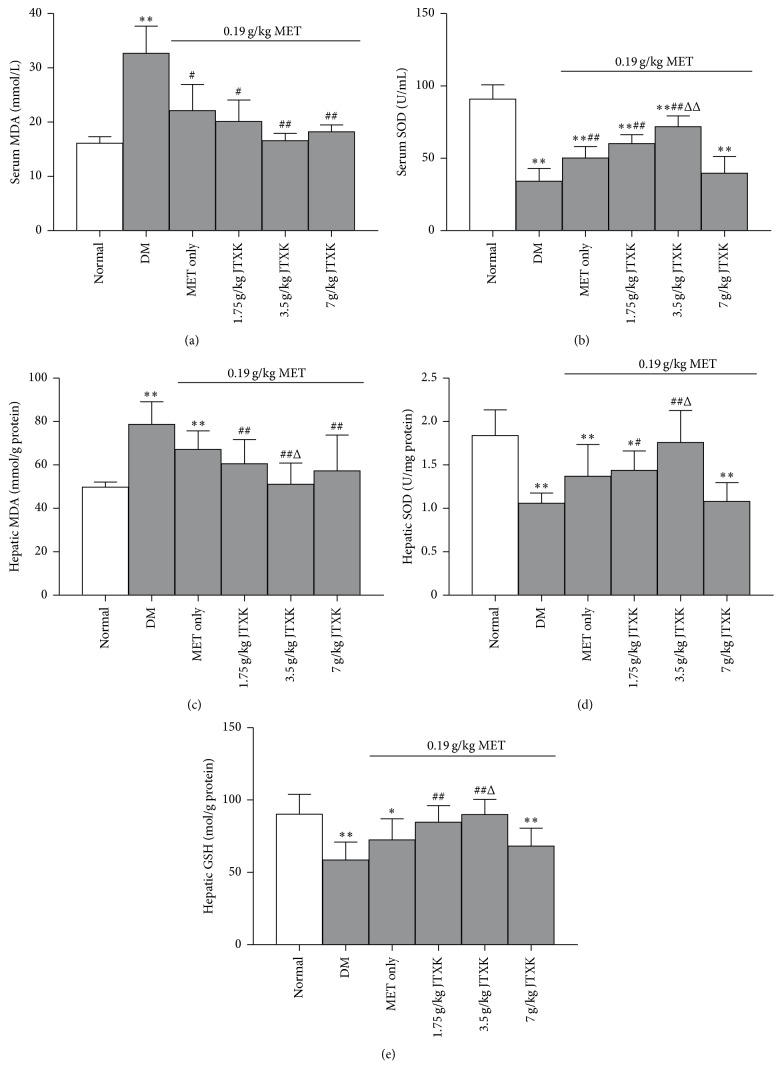
Effect of MET combined with JTXK on oxidative stress in DM mice. ^*∗*^ *p* < 0.05 and ^*∗∗*^ *p* < 0.01 versus the normal group; ^#^ *p* < 0.05 and ^##^ *p* < 0.01 versus the drug-untreated DM group; ^Δ^ *p* < 0.05 and ^ΔΔ^ *p* < 0.01 versus the MET alone group.

Serum (Figure 4(a)) and hepatic (Figure 4(c)) MDA levels were markedly elevated by 103% and 58.11%, respectively, in DM mice. However, serum SOD (Figure 4(b)), hepatic SOD (Figure 4(d)), and hepatic GSH (Figure 4(e)) levels were significantly reduced by 62.46, 42.44, and 35.08%, respectively, in DM mice, when compared with the normal animals. MET treatment decreased serum and hepatic MDA (by 32.28 and 14.77%, resp.) and increased serum and hepatic SOD (by 46.93 and 29.32%, resp.) and hepatic GSH (by 23.74%) compared with drug-untreated DM mice. MET combined with JTXK 3.5 g/kg was most effective in reducing serum and hepatic MDA levels (by 25.10 and 23.79%, resp.) and increasing serum SOD (by 43.09%), hepatic SOD (by 28.58%), and hepatic GSH (by 24.07%) contents, when compared with MET alone.

### 3.8. Effect of MET Combined with JTXK on Hepatic Histology in DM Mice

**Figure 5:**
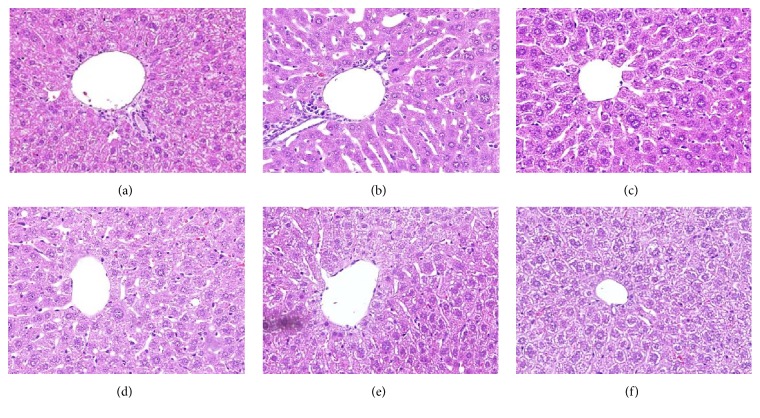
Effect of MET combined with JTXK on hepatic histology in DM mice: (a) normal mice; (b) drug-untreated DM mice; (c) DM mice treated with MET 0.19 g/kg; (d), (e), and (f) DM mice treated with MET 0.19 g/kg plus JTXK 1.75, 3.5, and 7 g/kg, respectively. Hematoxylin and Eosin (H&E) Staining (×20).

Light microscopic findings showed that there was no abnormal cell structure in the liver sections of normal mice (Figure 5(a)). Compared with normal liver, the liver architecture of DM mice showed an increased number of lipid droplets associated with hepatocytes hypertrophy, lymphocytes infiltration, sinusoidal space dilation, and microvascular steatosis (Figure 5(b)) in the mice fed with HFD. MET treatment showed a protective effect against DM induced liver injury, which was expressed as decreased sinusoidal space dilation and lymphocytes (Figure 5(c)). MET combined with JTXK (1.75, 3.5, and 7 g/kg) significantly reversed hepatotoxicity and hepatic steatosis caused by DM status in a dose-dependent manner (Figures 5(d), 5(e), and 5(f)).

### 3.9. Effect of MET Combined with JTXK on Body Weight in DM Mice

There was no difference in body weight between normal mice and DM mice. Treatment with MET alone had relatively little effect in lowering the body weight of DM mice. However, treatment with MET combined with JTXK lowered body weight during the period of medication compared with the MET treatment alone (Table 5).

## 4. Discussion

Currently, type 2 diabetes mellitus (T2DM) patients make up about 90% of all patients with DM [35, 36]. Present observations indicate that diabetes can be a driving force for NAFLD in terms of inflammation and oxidative stress [37, 38]. STZ is the most commonly used diabetogenic agent to establish diabetes animal model by destroying pancreatic *β* cells selectively, which can lead to insulin resistance and oxidative stress systemically [39–42]. HFD also associates with insulin resistance and adipocyte dysfunction; the high prevalence of NAFLD and insulin resistance among obese individuals reflect this relationship between HFD and NAFLD [43–45]. In this way, HFD fed animals with exposure to low dose of STZ are commonly used in scientific research for DM models. In the present study, DM mice model induced by HFD/STZ showed stable fasting hyperglycemia associated with impairment of glucose tolerance and elevation in FINS and glucagon, which indicated the glucose metabolism disorders. Meanwhile, metabolic abnormality of lipid such as hyperlipidemia and hepatic steatosis, liver injury, and oxidative stress status in both blood and liver were also observed in DM mice. All these results implied that DM associated with fatty liver in human was successfully mimicked by a mouse model induced by HFD intake and STZ injection.

Liver plays a vital role in the regulation of systemic glucose and lipid metabolism. In the setting of DM conditions, target organs such as liver, adipose tissue, and muscle tissue show insensitivity to the stimuli of insulin. At the same time, glucose utilization is reduced and adipose tissue lipolysis is enhanced, contributing to excessive systemic glucose and lipid levels. These changes are accompanied by compensative increased insulin secretion and hepatocellular energy alterations. Systemic lipids entered liver and increased hepatic fatty acid *β*-oxidation. When the mount of lipid exceeds the oxidative capacity, it will be deposited in the liver, referred to a condition as steatosis. As steatosis progresses, liver lobule and portal area are infiltrated with inflammatory cells and hepatocytes are filled with lipid droplets, resulting in steatohepatitis.

T2DM is a progressive disease which required lifestyle modifications and pharmacological interventions such as oral antihyperglycaemic agents and insulin injection [46–50]. However, some patients will not achieve their ideal glycaemic control until two oral antihyperglycaemic agents are administered. For some patients, sustained diabetes control is not achieved even after taking two agents in severe insulin resistance [51–54]. JTXK can improve the function of the liver, spleen, and kidney organ system and dispel pathogenic factors according to the theory of TCM. In our previous study, JTXK is effective in improving lipid metabolism, reducing DM symptoms and complications through regulating the activation of adenosine monophosphate activated protein kinase (AMPK), which is a regulator of energy metabolism and a key mechanism that brings about a wide range of metabolic benefits [55, 56]. Phosphorylated AMPK blocks SREBP1c, a transcription factor controlling enzymes involved in the fatty acid synthesis, contributes to lipid metabolism [55]. Currently, FBG, FINS, and OGTT levels are clinical parameters for definitely diagnosing DM. Here, they were used as the indicators for evaluating the effectiveness of the MET and JTXK plus MET antidiabetic activity in DM mice. Treating mice with combination with MET and JTXK significantly reduced FBG, FINS, and glucagon levels and partly restored glucose tolerance compared with MET alone. These findings suggested that combination treatment with MET and JTXK (3.5 g/kg) effectively reversed the disturbance of glucose and lipid metabolism and oxidative stress status in DM mice. It is well known that MET, an insulin sensitizer, inhibits hepatic glucose production via decreasing gluconeogenesis, increasing glycogenolysis, and diverting fatty acids from TG to mitochondrial beta oxidation [57]. In the present study, it was found that JTXK treatment could improve the antidiabetic effect of MET. It means that JTXK affects MET-induced glucose and lipid metabolism through the same pathways. Further studies are needed.

It is well established that serum aminotransferase activity is widely adopted as sensitive biomarker of liver damage in both clinic and animal experiment. In the present study, increased serum ALT and AST activities and decreased sinusoidal space dilation and lymphocytes in liver histology are commonly regarded as signs of credible hepatic injury. Treatment with MET and JTXK altered serum and hepatic lipid contents and ameliorated hepatic injury in DM mice. Hepatic lipid-lowering effect of MET alone and combined with JTXK was shown in a dose-dependent manner in both serum and liver. Some studies have reported that administration of MET resulted in improving aminotransferase levels, while others have not found this effect [58–62]. In the current study, it was found MET treatment alone lowered the serum ALT and AST activities, which was enhanced by combination with JTXK 3.5 g/kg, in DM mice. However, large dose of JTXK (7 g/kg) eliminated ALT- and AST-lowering effect of MET.

Oxidative stress plays a key role in the development of DM and chronic complications in DM [63]. DM may induce hepatic MDA formation and lipid peroxidation, which can affect the fluidity and permeability of hepatocytes membrane and lead to cellular damage [64]. The reduced activities of antioxidative enzymes (SOD, GSH, etc.) also indicated the insufficient ability against oxidative stress. In the present study, treatment with MET and JTXK improved the antioxidant ability and restored their activities near to normal group. Combination therapy was more effective than given MET alone, except large dose of JTXK (7 g/kg) combination.

In conclusion, the current study revealed that JTXK, a Chinese herbal medicine formula, treatment for 4 weeks could promote the improvement of MET on the serum and liver glucose and lipid metabolism, as well as insulin sensitivity in HFD and low dose STZ induced DM mice. The hepatoprotective activity and antioxidative activity of MET against DM were accelerated by the combination with JTXK. Combination therapy with proper dose of JTXK (classic Chinese herbal formula) and MET (chemical drug) may represent a good strategy for the management of the patient with DM.

## Figures and Tables

**Table 1:** Effect of MET combined with JTXK on serum FBG levels in DM mice.

Groups	Dose (g/kg)	FBG (mmol/L)		
		Before treatment	2 weeks of treatment	4 weeks of treatment
Normal	—	5.98 ± 0.69	6.03 ± 0.78	5.95 ± 0.60
DM	—	27.13 ± 2.31^*∗∗*^	26.78 ± 3.05^*∗∗*^	26.34 ± 3.17^*∗∗*^
MET	0.19	28.70 ± 2.90^*∗∗*^	21.92 ± 4.56^*∗∗*^	15.97 ± 2.46^*∗∗*##^
MET/JTXK	0.19/1.75	26.52 ± 3.65^*∗∗*^	20.03 ± 3.01^*∗∗*#^	15.53 ± 1.87^*∗∗*##^
	0.19/3.5	28.65 ± 4.84^*∗∗*^	16.97 ± 3.01^*∗∗*##^	11.12 ± 3.13^*∗∗*##ΔΔ^
	0.19/7	27.78 ± 2.05^*∗∗*^	16.53 ± 4.12^*∗*#^	13.50 ± 3.24^*∗∗*##^

^*∗*^ *p* < 0.05 and ^*∗∗*^ *p* < 0.01 versus the normal group; ^#^ *p* < 0.05 and ^##^ *p* < 0.01 versus the drug-untreated DM group; ^ΔΔ^ *p* < 0.01 versus the MET alone group.

**Table 2:** Effect of MET combined with JTXK on OGTT levels in DM mice.

Groups	Dose	Blood glucose (mmol/L) after oral glucose gavage			
	(g/kg)	0 min	30 min	60 min	120 min
Normal	—	6.13 ± 0.65	13.03 ± 0.60	10.17 ± 1.92	7.17 ± 0.76
DM	—	26.90 ± 1.22^*∗∗*^	33.30 ± 0.00^*∗∗*^	32.33 ± 1.00^*∗∗*^	30.40 ± 1.25^*∗∗*^
MET	0.19	17.67 ± 1.10^*∗∗*##^	31.57 ± 1.55^*∗∗*^	27.30 ± 1.40^*∗∗*#^	20.93 ± 1.26^*∗∗*##^
MET/JTXK	0.19/1.75	17.97 ± 2.21^*∗∗*##^	30.90 ± 2.12^*∗∗*^	28.97 ± 2.10^*∗∗*^	20.23 ± 2.46^*∗∗*##^
	0.19/3.5	11.40 ± 3.32^*∗*##ΔΔ^	27.90 ± 4.07^*∗∗*^	24.80 ± 4.44^*∗∗*##^	12.90 ± 2.36^*∗∗*##ΔΔ^
	0.19/7	12.60 ± 3.41^*∗∗*##Δ^	29.90 ± 2.95^*∗∗*^	26.60 ± 2.51^*∗∗*#^	21.10 ± 2.70^*∗∗*##^

^*∗*^ *p* < 0.05 and ^*∗∗*^ *p* < 0.01 versus the normal group; ^#^ *p* < 0.05 and ^##^ *p* < 0.01 versus drug-untreated DM group; ^Δ^ *p* < 0.05 and ^ΔΔ^ *p* < 0.01 versus the MET alone group.

**Table 3:** Effect of MET combined with JTXK on serum lipids in DM mice.

Groups	Dose (g/kg)	TG	TC	HDL	LDL
		(mmol/L)	(mmol/L)	(mmol/L)	(mmol/L)
Normal	—	0.64 ± 0.10	2.65 ± 0.16	4.70 ± 0.31	0.25 ± 0.03
DM	—	3.42 ± 1.06^*∗*^	8.87 ± 1.06^*∗∗*^	2.64 ± 0.20^*∗∗*^	1.02 ± 0.30^*∗*^
MET	0.19	2.10 ± 0.30^*∗∗*^	5.62 ± 0.66^*∗∗*##^	4.17 ± 0.66^#^	0.80 ± 0.18^*∗∗*^
MET/JTXK	0.19/1.75	1.84 ± 0.18^*∗∗*^	5.25 ± 1.00^*∗∗*##^	5.25 ± 0.77^##^	0.74 ± 0.20^*∗*^
	0.19/3.5	1.68 ± 0.23^*∗∗*^	4.84 ± 0.70^*∗∗*##^	4.48 ± 0.34^##^	0.72 ± 0.12^*∗∗*^
	0.19/7	1.29 ± 0.45^#^	4.09 ± 0.90^*∗∗*##ΔΔ^	4.79 ± 1.17	0.68 ± 0.08^*∗∗*^

^*∗*^ *p* < 0.05 and ^*∗∗*^ *p* < 0.01 versus the normal group; ^#^ *p* < 0.05 and  ^##^ *p* < 0.01 versus the drug-untreated DM group; ^ΔΔ^ *p* < 0.01 versus the MET alone group.

**Table 4:** Effect of MET combined with JTXK on hepatic function and mass in DM mice.

Groups	Dose (g/kg)	ALT activity	AST activity	Hepatic weight	Hepatic index
		(U/L)	(U/L)	(g)	(%)
Normal	—	28.33 ± 4.93	23.87 ± 4.06	2.18 ± 0.20	5.02 ± 0.45
DM	—	92.73 ± 13.64^*∗∗*^	77.61 ± 5.33^*∗∗*^	4.22 ± 0.45^*∗∗*^	9.77 ± 0.80^*∗∗*^
MET	0.19	61.47 ± 9.39^*∗∗*#^	48.37 ± 5.32^*∗∗*##^	3.27 ± 0.42^*∗∗*##^	8.01 ± 0.89^*∗∗*##^
MET/JTXK	0.19/1.75	48.50 ± 7.34^*∗∗*##^	42.93 ± 7.00^*∗∗*##^	2.94 ± 0.44^*∗∗*##^	7.69 ± 1.18^*∗∗*##^
	0.19/3.5	41.80 ± 5.81^*∗*##Δ^	36.47 ± 3.40^*∗∗*##ΔΔ^	2.44 ± 0.40^##ΔΔ^	6.59 ± 1.08^*∗*##ΔΔ^
	0.19/7	78.70 ± 6.85^*∗∗*^	69.07 ± 7.81^*∗∗*#ΔΔ^	2.36 ± 0.32^##ΔΔ^	6.27 ± 1.01^##ΔΔ^

^*∗*^ *p* < 0.05 and ^*∗∗*^ *p* < 0.01 versus the normal group; ^#^ *p* < 0.05 and ^##^ *p* < 0.01 versus the drug-untreated DM group; ^Δ^ *p* < 0.05 and ^ΔΔ^ *p* < 0.01 versus the MET alone group.

**Table 5:** Effect of MET combined with JTXK on body weight in DM mice.

Groups	Dose	Body weight (g) after treatment with drugs				
	(g/kg)	Week 0	Week 1	Week 2	Week 3	Week 4
Normal	—	41.50 ± 1.58	42.22 ± 1.80	42.33 ± 1.93	43.42 ± 2.04	43.45 ± 2.09
DM	—	42.92 ± 2.01	43.13 ± 0.82	43.13 ± 0.97	44.53 ± 1.12	43.15 ± 1.55
MET	0.19	41.18 ± 1.80	41.83 ± 1.60	40.93 ± 2.35^#^	41.58 ± 2.69^#^	40.88 ± 2.57^#^
MET/JTXK	0.19/1.75	40.93 ± 1.38	39.68 ± 1.88^##Δ^	38.52 ± 2.21^##Δ^	38.65 ± 1.62^*∗*##Δ^	38.32 ± 2.01^*∗*##Δ^
	0.19/3.5	42.02 ± 1.67	40.25 ± 1.59^##^	37.87 ± 1.54^*∗∗*##ΔΔ^	36.93 ± 1.59^*∗∗*##ΔΔ^	37.12 ± 0.86^*∗∗*##ΔΔ^
	0.19/7	41.32 ± 1.65	38.85 ± 2.15^##ΔΔ^	38.30 ± 1.53^*∗∗*##Δ^	38.87 ± 2.00^*∗*##Δ^	37.80 ± 1.68^*∗∗*##ΔΔ^

^*∗*^ *p* < 0.05 and ^*∗∗*^ *p* < 0.01 versus the week 0; ^#^ *p* < 0.05 and ^##^ *p* < 0.01 versus the drug-untreated DM group.; ^Δ^ *p* < 0.05 and ^ΔΔ^ *p* < 0.01 versus the MET alone group.
